# Meloxicam Inhibits Hepatocellular Carcinoma Progression and Enhances the Sensitivity of Immunotherapy via the MicroRNA-200/PD-L1 Pathway

**DOI:** 10.1155/2022/4598573

**Published:** 2022-02-21

**Authors:** Sun Guangshun, Sun Guoqiang, Chen Xin, Kong Xiangyi, Zheng Wubin, Li Zhitao, Zheng Zhiying, Cao Hongyong, Lv Chengyu, Xia Yongxiang, Tang Weiwei

**Affiliations:** ^1^Department of General Surgery, Nanjing First Hospital, Nanjing Medical University, Nanjing, Jiangsu, China; ^2^Hepatobiliary/Liver Transplantation Center, The First Affiliated Hospital of Nanjing Medical University, Key Laboratory of Living Donor Transplantation, Chinese Academy of Medical Sciences, Nanjing, Jiangsu, China; ^3^Department of Anesthesiology, The First Affiliated Hospital of Nanjing Medical University, Nanjing 210000, Jiangsu, China

## Abstract

**Background:**

Hepatocellular carcinoma (HCC) has become the sixth most common cancer and the third leading cause of cancer death in the world. Although the research achievements of tumor immunotherapy have made great progress, especially the combination of immune targeted therapy has achieved good curative effect in HCC, but only a few patients are suitable for it and benefit from it. Therefore, there is an urgent need to find new effective drugs to treat HCC or to enhance the sensitivity of immunotherapy.

**Methods:**

Meloxicam, a COX2 inhibitor with strong anti-HCC potential, was screened from 800 small molecules approved by FDA. The effect of meloxicam on the proliferation, invasion, and migration of HCC cell lines was evaluated by cell phenotype analysis. The Human Protein Atlas database and the TISCH database were used to analyze COX2 data in single cells, and the TISIDB database was used to analyze the correlation of COX2 with immune function. The real-time quantitative polymerase chain reaction (qRT-PCR) and western blot were used to evaluate the level of PD-L1 and CD155 in HCC cell lines treated with meloxicam and further explore its possible mechanism. In vivo experiments were applied to verify the effect of meloxicam combined with anti-PD1 therapy on HCC tumor growth in mice.

**Results:**

Meloxicam can significantly inhibit the proliferation, invasion, and migration of HCC cells. The TISIDB database indicated that the COX2 was strongly associated with immunoinhibitors and immunostimulators. Meloxicam upregulated the level of PD-L1 in HCC cell lines and animal models. In terms of mechanism, meloxicam inhibited microRNA-200, thereby upregulating PD-L1. In vitro experiments showed that both meloxicam and anti-PD1 had inhibitory effects on the growth of HCC tumors. Compared with meloxicam and anti-PD1 alone, the combination therapy showed stronger antitumor properties. Immunohistochemical analysis confirmed that meloxicam enhanced the antitumor immune activity in the tumor microenvironment.

**Conclusion:**

Our study showed meloxicam inhibited HCC progression and enhanced the sensitivity of immunotherapy via the microRNA-200/PD-L1 pathway.

## 1. Introduction

Hepatocellular carcinoma (HCC) is the most common primary tumor of the liver, accounting for more than 90% of primary liver tumors. Approximately 80% of patients diagnosed with liver cirrhosis will develop HCC [1]. Existing statistics indicate that HCC is the fifth most common tumor in the world, and it ranks second in the cause of tumor deaths in men [2]. Studies have shown that the five-year survival rate of HCC is only 18%, which is currently second only to the king of cancers, pancreatic cancer [3]. There are many factors that can lead to the occurrence of HCC, such as viral hepatitis (hepatitis B and C)/fatty liver. Current treatments for HCC include surgical resection, liver transplantation, tumor ablation, transarterial therapies, and systemic chemotherapy. Among them, surgical treatment is the most important treatment for early-onset HCC [4]. Although there are so many treatments for HCC, the overall survival of patients is still unsatisfactory. Therefore, more innovative treatment methods need to be developed.

One of the difficulties of cancer treatment lies in its complex immune evasion mechanism, and one of the key mechanisms is the expression of multiple immunosuppressive ligands on the surface of cancer cells. The most representative ligand is programmed cell death 1 ligand 1 (PD-L1). PD-L1 can bind to the programmed cell death protein 1 (PD1) receptor on the surface of T cells, thereby inhibiting the proliferation of T cells/the production and release of cytokines and its cytolytic activity. Therefore, blocking and inhibiting PD-L1 or PD1 can restore the immune activity of T cells to a certain extent and then restore the immune surveillance and immune attack on tumors [5, 6]. Currently, studies on PD-L1 and PD1 have attracted more and more attention. Multiple checkpoint blocking immunotherapy have been developed to treat a variety of cancers including HCC. According to a study in 2020, the PD-L1 monoclonal antibody atezolizumab combined with bevacizumab in the treatment of advanced HCC was significantly better than traditional sorafenib in terms of overall survival and progression-free survival [7], which gradually established the status of immune combined targeted therapy in the treatment of HCC.

Although a variety of checkpoint inhibition treatments have achieved impressive results, but clinically, factors such as PD1 monoclonal antibody resistance mean that these immunotherapies can only benefit a small number of patients. Therefore, the crosstalk between conventional and targeted anticancer therapies combined with checkpoint suppression therapies may lead to more effective combined treatments for cancer. In this study, we screened more than 800 kinds of FDA drug library and fortunately found that meloxicam (COX2 inhibitor) is a drug that can effectively inhibit the growth and proliferation of HCC cells, and its anticancer effect has been confirmed in a variety of cancers preciously. Cyclooxygenase-2 (COX2) is a rate-limiting enzyme that is mainly involved in the synthesis of prostaglandin (PG). Past studies have shown that it is overexpressed in many cancers including HCC. And it is regarded as a potential anticancer target [8, 9]. There is already evidence that COX2 can be induced by hypoxia and other conditions. COX2 can promote cell growth and angiogenesis and also slow cell apoptosis [10, 11]. However, how and whether meloxicam plays a role in cancer-related immunity remains unclear, so this study will focus on this issue.

## 2. Methods and Materials

### 2.1. Drug Screening

The FDA drug library including meloxicam was purchased from Selleck Chemicals LLC (USA). The Cell Counting Kit-8 (CCK-8) test experiment was used to screen more than 800 drugs. YY8103 and Hep3B cells were used to first undergo the dosing and incubation process at 37°C.

### 2.2. Cell Cultures

In the study, YY8103, Hep3B, and Hep1-6 cells were cultured in DMEM medium (BI, USA), which also contained 10% fetal bovine serum (FBS) (Gibco, USA), 5% streptomycin, and penicillin, incubated in a CO_2_ chamber at 37°C. Meloxicam comes from Horizon Corporation (Nanjing, China). The YY8103 and Hep3B cell lines used were treated with meloxicam at 10 µM for 48 h. The cells obtained in this study were used in subsequent related experiments.

### 2.3. Transwell Assay

According to the manufacturer's instructions, we inoculated processed YY8103 and Hep3B cells into 200 *μ*l serum-free DMEM medium in the upper chamber. The transwell room (Corning, USA) accepted the paving process, used Matrigel mix (BD Biosciences, USA) for the invasion test process, and did not use the Matrigel mix for the migration test. DMEM medium and 10% FBS are introduced into the bottom chamber to become HCC cytochemical attractants. When the 48-hour incubation process is completed, the upper chamber is fixed and then stained with crystal violet (Kagan, China) for 15 minutes. For the visualization process, the cell line received the photo and counting process in three fields.

### 2.4. Wound Healing Assay

YY8103 and Hep3B cells undergo a drug treatment process when they are seeded on a 6-well culture plate. Using a standard 20 *μ*l pipette tip can eliminate artificial linear wounds on a single layer of confluent cells. Remove free-floating cells and debris. Incubate the plate at 37°C in DMEM medium containing 10% FBS, 5% streptomycin, and penicillin. Record the width of the scratch under an inverted microscope, and then take pictures at 0, 24, and 48 hours.

### 2.5. Cell Proliferation Assay

We inoculated 1000 cancer cells in 96-well plates and treated them with 10 *μ*L CCK-8 solution (RiboBio, China) at 0 h, 24 h, 48 h, and 72 h. According to the manufacturer's instructions (Synergy, USA), cell absorbance is measured at various time points at 450 nm using a microplate reading element. We spread 5 × 10^4^ cancer cells in 24-well plates and cultured the cells for 24 hours. Cell lines were incubated with a 50 mmol/L EdU solution for 2 h and fixed with 4% paraformaldehyde. According to the manufacturer's agreement, cell lines were treated with Apollo Dye Solution and Hoechst SEAL. EdU cell lines were captured and counted under an Olympus FSX100 microscope (Olympus, Japan). During the plate clone formation experiment, the transfected cells were seeded in a 6-well plate at a density of 1000 cells per well, and then cultured in DMEM medium containing 10% FBS. After 10 days, the cells received a fixation based on the use of methanol and then stained with GIMSA. Finally, the colonies are imaged and counted.

### 2.6. COX2 Expression Level in Single Cells and Immune Analysis

The Human Protein Atlas database and TISCH database were used to analyze the expression of COX2 in single liver cell and single HCC cell. The TISIDB database was used to analyze the correlation between COX2 expression and immune cells.

### 2.7. RNA Extraction and Real-Time Quantitative Polymerase Chain Reaction (qRT-PCR)

We used TRIzol reagent (Invitrogen, USA) to extract total RNA from precultured and transfected HCC cell lines. After induction and RNA quantification and quality testing, the total RNA was reverse transcribed into complementary DNA using the PrimeScript RT kit (Takara, China) under the recommended conditions. According to the manufacturer's instructions, qRT-PCR was performed on an ABI 7500 instrument in triplicate. The primer sequences used in this experiment are microRNA-200, microRNA-513, PD-L1, CD155, and Glyceraldehyde 3-phosphate dehydrogenase (GAPDH). GAPDH is used to normalize mRNA expression levels, while U6 is used for miRNA.

### 2.8. Mice Model

The animal management committee of Nanjing Medical University approved the animal experiment, and all experiment procedures and animal caring conformed to the institutional ethics directions for animal-related experiments. We used 5-week-old male C57BL/6 mice to construct tumor xenograft models. The mice were randomly divided into 4 groups for intraperitoneal injection. The groups were PBS, meloxicam, anti-PD1, and meloxicam + anti-PD1 (*n* = 5 for the respective groups). 1 × 10^6^ Hep1-6 cells were inoculated into right groin of C57BL/6 mice. A 5 mg/kg meloxicam intraperitoneal injection was made for the meloxicam group every four days. A 6.6 mg/kg intraperitoneal injection was made for the anti-PD1 group on the eighth day and once per four days thereafter. After 16 days, the mice were killed, and the tumor tissue was taken out for weighing and immunohistochemical analysis.

### 2.9. Immunohistochemical Staining

Tumor tissues from mice were embedded in paraffin blocks for immunohistochemical staining and analysis. Tissue sections were deparaffinized and hydrated. Sections were incubated with 3% H2O2 for 10 min and then incubated at 4°C with primary antibodies (CD4, CD8, Ki67, COX2, PD1, and PD-L1) overnight. A secondary antibody was added and incubated at 37°C for 15 min. Tissue sections were stained using diaminobenzidine and hematoxylin. Finally, sections were dehydrated and covered with glass slides. All tissue sections were photographed using a microscope camera and analyzed using the TissueFAXS Viewer software program.

### 2.10. Statistical Analysis

The continuing information received the comparative analysis by performing one individual t-testing process of the two groups. A statistics-related analysis process was performed and presented graphically in GraphPad Prism 8.0. A *P* value of 0.05 was considered to be statistically significant.

## 3. Results

### 3.1. Pharmacologically Active Drug Screening Confirmed That Meloxicam Had Anti-HCC Activity

**Figure 1:**
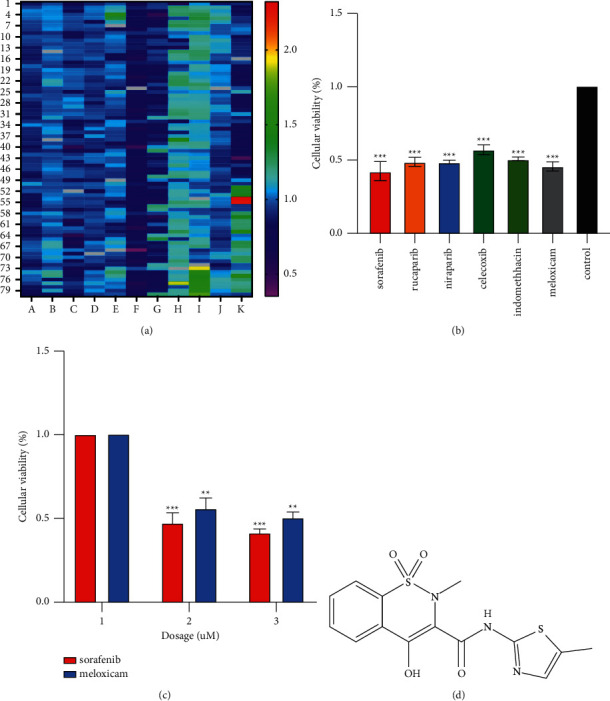
Results of screening drugs for anti-HCC ability of 800 compounds approved by the US FDA. (a) Results of screening 800 drugs using CCK8. (b) Results of rescreening several drugs with better efficacy. (c) The effect of meloxicam and sorafenib on the activity of HCC cells at different concentrations. (d) Chemical formula of meloxicam.

In order to screen new drugs with anti-HCC efficacy, we used the CCK8 method to determine more than 800 drugs against the YY8103 cell line, and finally determined that meloxicam had relatively good anti-HCC properties (Figure 1(a) and Supplementary Table 1). The chemical formula of meloxicam is shown in Figure 1(c). We screened the top six drugs with the best effects and tested their inhibitory effects on HCC cells at a level of 10 µM. We found that meloxicam had a relatively good performance (Figure 1(b)). We further compared the effects of meloxicam and the mainstream anti-HCC drug sorafenib on HCC cells at different concentration levels. The results showed that the performance of meloxicam was satisfactory (Figure 1(c)).

### 3.2. Meloxicam Effectively Inhibited the Proliferation, Migration, and Invasion of HCC Cells

**Figure 2:**
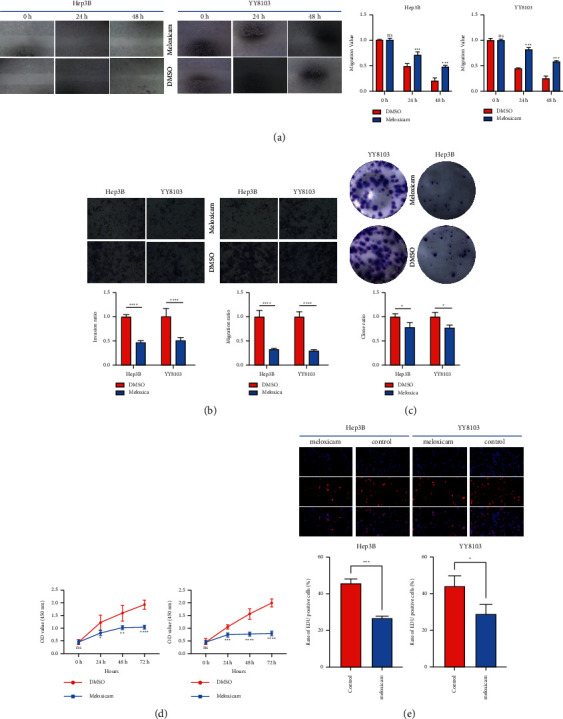
Treatment of HCC cell lines with meloxicam could cause significant inhibitory effects on it. (a) The scratch test confirmed that meloxicam inhibited the invasion and metastasis ability of HCC cell lines. (b) After treatment with meloxicam, the migration and invasion of HCC cells were also inhibited. (c–e) The plate cloning experiment, the CCK8 experiment, and the EDU experiment confirmed that after treatment with meloxicam, the proliferation of HCC was effectively inhibited. ^*∗*^*P* < 0.05; ^*∗∗*^*P* < 0.01; ^*∗∗∗*^*P* < 0.001; ^*∗∗∗∗*^*P* < 0.0001.

In order to further verify the role of meloxicam in HCC cell lines, we added 10 µM meloxicam to the experimental group's culture medium, and added the same amount of DMSO to the control group, and verified its effect through different assays. Scratch experiments confirmed that after adding 10 µM meloxicam, cell migration capabilities were significantly reduced (Figure 2(a)). The plate cloning experiment analysis and the result of CCK and the EDU assays show that the proliferation rate of HCC cell lines with 10 µM meloxicam was significantly reduced (Figures 2(c)–2(e)). In addition to the abovementioned experiments, we also performed the transwell experiment to verify the effect of meloxicam on the migration and invasion of HCC cell lines (Figure 2(b)). In summary, meloxicam could effectively inhibit the proliferation, migration, and invasion of HCC cells.

### 3.3. Expression Data of COX2 at the Single Cell Level of HCC

**Figure 3:**
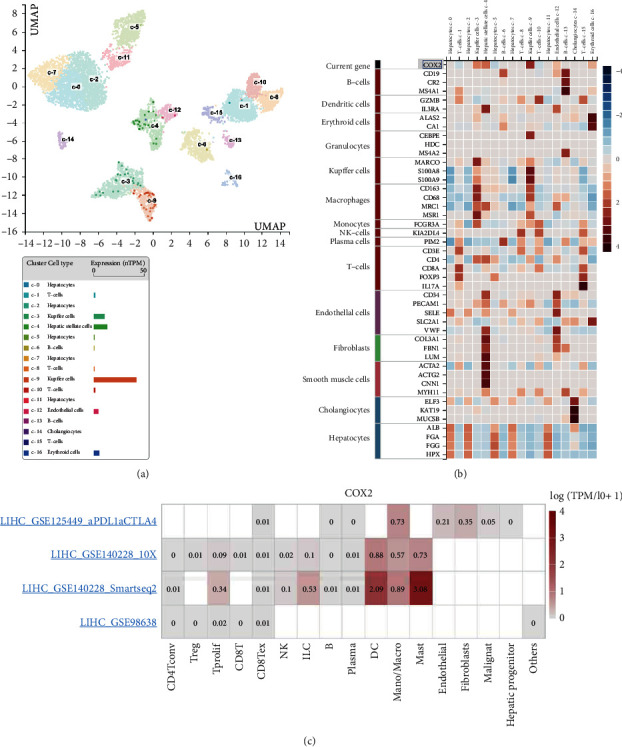
Expression data of COX2 at the single cell level of hepatocellular carcinoma. (a, b) The expression of COX2 in single liver cell. (c) The expression of COX2 in a single HCC cell.

We used the Human Protein Atlas database and TISCH database to analyze single-cell level data of COX2 in the liver. The result indicates that COX2 is mainly expressed in Kupffer cells, hepatic stellate cells, endothelial cells, and T cells in the liver (Figures 3(a) and 3(b)). The TISCH database including LIHC_GSE125449_aPDL1aCTLA4, LIHC_GSE140228_10X, LIHC_GSE140228_Smartseq2, and LIHC_GSE98638 showed that in HCC, COX2 was mainly expressed in mast cells, mono/macro cells, and dendritic cells (DC) (Figure 3(c)). Those results indicated that COX2 might be associated with immune responses in HCC.

### 3.4. Expression of COX2 Was Correlated with Immune-Related Factors

**Figure 4:**
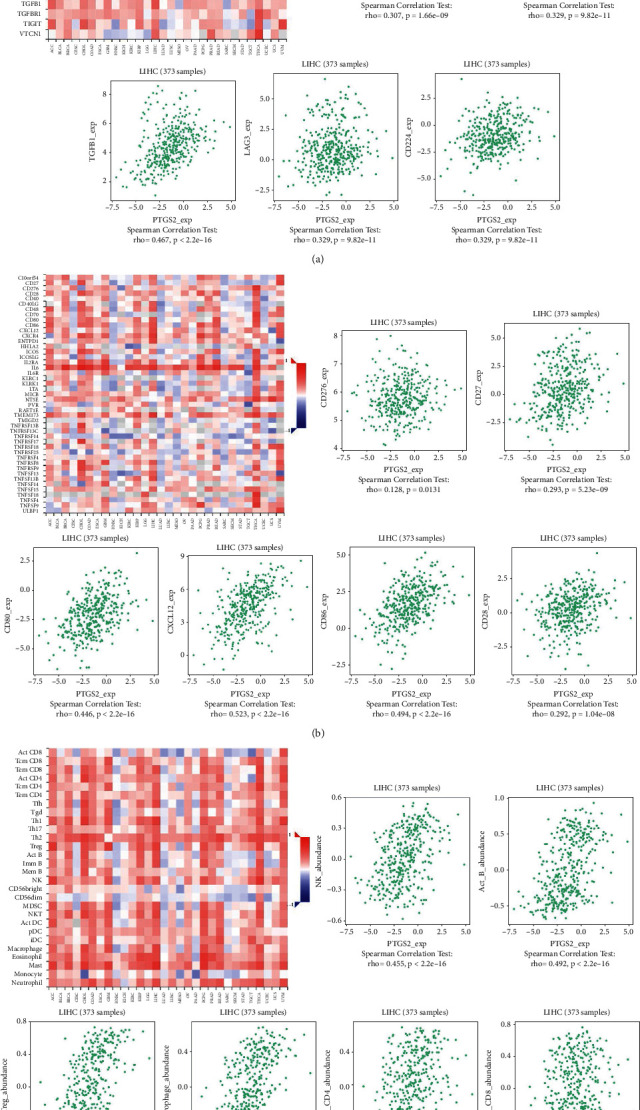
Correlations between COX2 expression and three cancer-related immune factor types. (a) Correlation between COX2 expression and immunoinhibitors in HCC. (b) Correlation between COX2 expression and immunostimulator in HCC. (c) Correlation between COX2 expression and lymphocyte in HCC.

Previous studies have confirmed the connection between tumor development and immune factors. We further explored the relationship between COX2 expression and some immune factors in different cancers using the TISIDB database. The result indicated that the expression of COX2 in spearman was associated with immunoinhibitors, immunostimulators, and lymphocytes (Figures 4(a)–4(c)). These results further indicated the potential effect of COX2 in the immune response.

### 3.5. Meloxicam Could Enhance the Expression of PD-L1 through Interaction with MicroRNA-200

**Figure 5:**
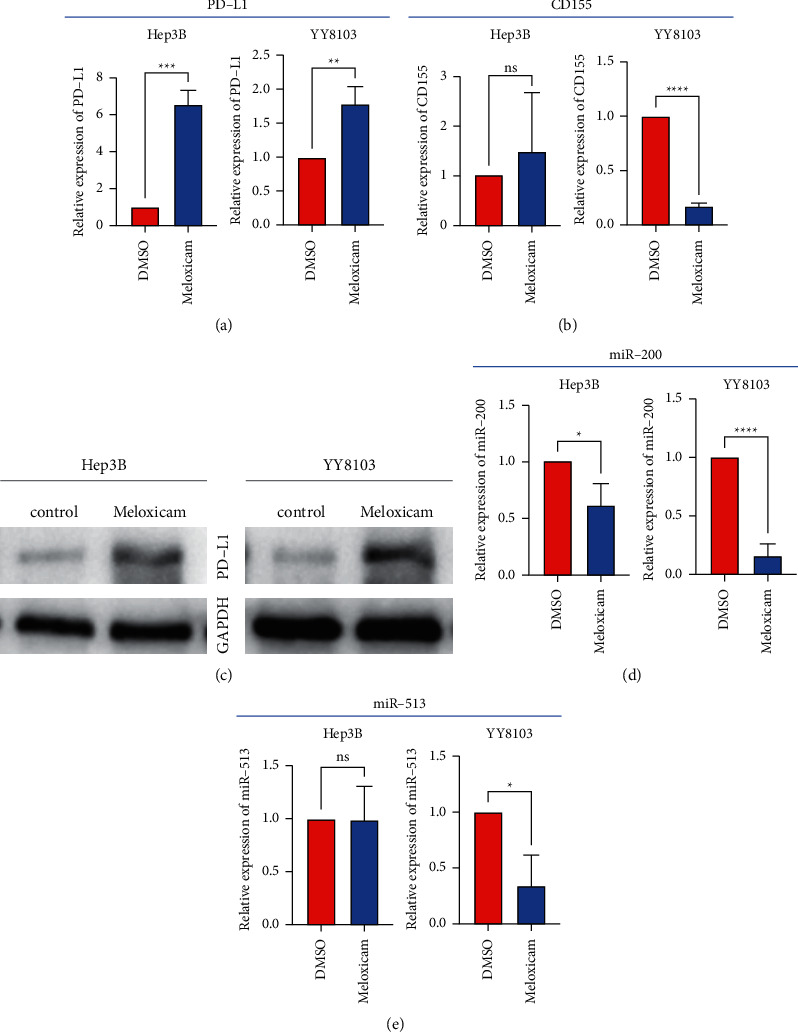
Results of qRT-PCR and western blot. (a) After treatment with meloxicam, the expression of PD-L1 on the surface of YY8103 and Hep3B cells increased. (b) After treatment with meloxicam, the change of expression of CD155 on the surface of YY8103 and Hep3B cells. (c) The result of western blot after treatment with meloxicam. (d) After treatment with meloxicam, the expression of microRNA-200 of YY8103 and Hep3B cells downregulated. (e) After treatment with meloxicam, the expression of microRNA-513 of Hep3B cells. ^*∗*^*P* < 0.05; ^*∗∗*^*P* < 0.01; ^*∗∗∗*^*P* < 0.001; ^*∗∗∗∗*^*P* < 0.0001.

In order to further explore the role of meloxicam in inhibiting the occurrence and development of HCC, we selected candidate genes PD-L1 and CD155 related to tumor immunity to explore the relationship between them. We used qRT-PCR to compare the expression of PD-L1 and CD155 on the surface of Hep3B and YY8103 cell lines after adding 10 µM meloxicam. To our surprise, the expression of PD-L1 instead of CD155 on the cell line surface increased significantly (Figure 5(a) and 5(b)). Western Blot future validated this result (Figure 5(c)). Studies have shown that the expression of PD-L1 on the cell surface is regulated by many factors. After consulting a large amount of literature, we chose microRNA-200 and microRNA-513 as testing objects. The qRT-PCR results indicated that after adding meloxicam in Hep3B and YY8103 cells, the expression of microRNA-200 was downregulated, and the correlation with the expression of microRNA-513 was not obvious (Figures 5(d) and 5(e)). Studies have confirmed that downregulation of microRNA-200 could upregulate the expression of PD-L1 in breast cancer [12], which suggested that meloxicam may regulate the expression of PD-L1 through its interaction with microRNA-200.

### 3.6. Meloxicam Combined with PD1 Monoclonal Antibody Could Inhibit the Growth of HCC In Vivo

**Figure 6:**
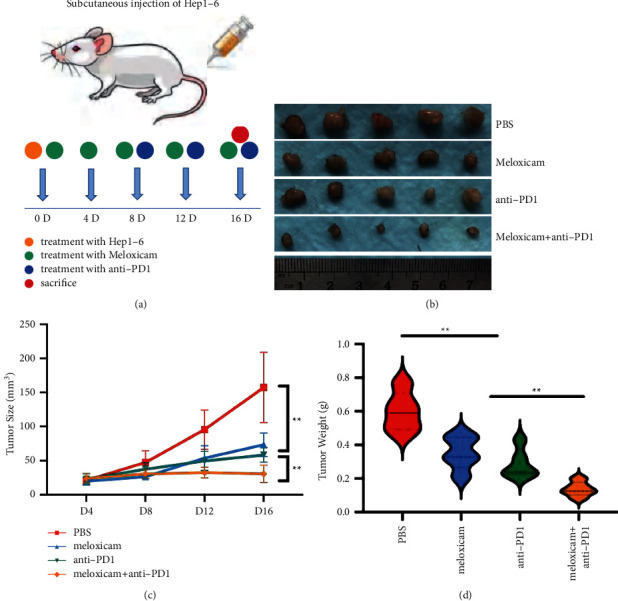
Meloxicam combined with PD1 monoclonal antibody could inhibit the growth of HCC in vivo. (a) Mice model injection illustration. (b) Picture display of the respective group of tumors. (c) The tumor volumes were measured every 4 days. (d) The relative weights of tumors were evaluated. ^*∗∗∗*^*P* < 0.001 and ^*∗∗∗∗*^*P* < 0.0001.

**Figure 7:**
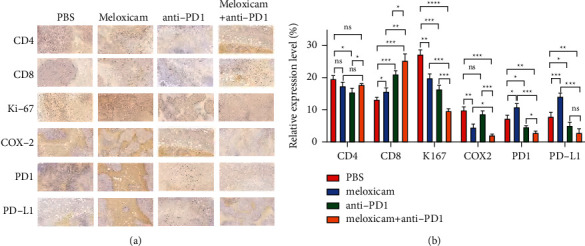
Result of immunohistochemical analysis. (a, b) Immunohistochemical analysis showed that there were significant differences in the staining of CD4, CD8, Ki67, etc. between the control group and the combination group.

**Figure 8:**
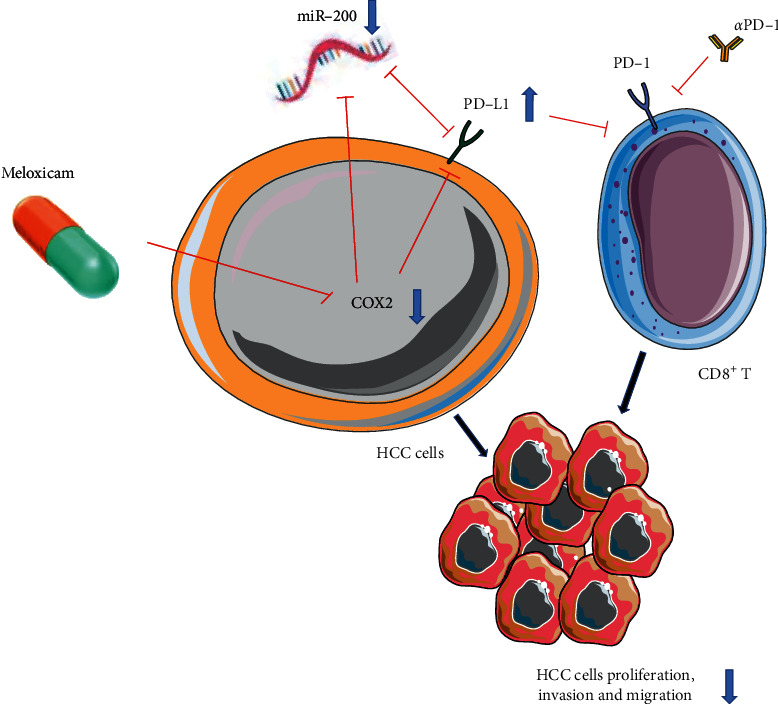
Schematic showing that meloxicam inhibits HCC and increases the efficacy of *α*PD-1 in the treatment of HCC.

We used 5-week-old male C57BL/6 mice to construct tumor xenograft models. The mice were randomly divided into 4 groups, with five mice in each group. The control group was injected with 100 µl PBS intraperitoneally. The “meloxicam” group was intraperitoneally injected with meloxicam 5 mg/kg, and the “anti-PD1” group was intraperitoneally injected with PD1 monoclonal antibody 6.6 mg/kg. After 16 days, all mice participating in the experiment were sacrificed, and tumors were collected for size and weight measurement (Figure 6(a)). The results showed that compared with the control group, the meloxicam group/anti-PD1 group/combination group's mice subcutaneous tumor volume was significantly reduced, and the weight was significantly reduced as well. Compared with the meloxicam group/anti-PD1 group, the subcutaneous tumor volume of mice in the combination group was significantly reduced and the weight was significantly reduced (Figures 6(b)–6(d)). Given the immunohistochemical results, the expression of CD4 in each group showed no significant difference. Compared with the PBS group, the meloxicam group significantly promoted the expression of CD8 and PD-L1, suggesting that the addition of meloxicam activated the immune function of HCC. When meloxicam was combined with anti-PD1, the expression of CD8 was significantly increased, and the expression of Ki-67, PD-L1, and PD1 was significantly decreased (Figures 7(a) and 7(b)). These differences suggested that the combination of meloxicam and PD1 monoclonal antibody had a strong therapeutic potential for HCC therapy in vivo. Finally, we drew a schematic showing that meloxicam inhibited HCC and increased the efficacy of anti-PD1 in the treatment of HCC (Figure 8).

## 4. Discussion

With the deepening of our research, we found that COX2 plays an important role in a variety of tumors, and it plays a role in different stages of tumorigenesis and development [13]. For example, COX2 is an inflammatory mediator of cancer and can inhibit the apoptosis of cancer cells [14–16]. COX2 participates in the formation of tumor blood vessels and can induce the activity of tumor stem cells [17–19].

Existing studies have found a variety of mechanism pathways to help COX2 achieve the abovementioned functions, including sirtuin (SIRT)/COX2 [20], NF-*κβ*/COX2 [21], COX2/STAT3 [22], PI3K/AKT/COX2 [23], COX-2/hypoxia inducible factor (HIF) [22], and other popular channels. The abovementioned studies have confirmed the important role of COX2 in the occurrence and development of cancer. Our research also confirmed the anti-HCC effect of the COX2 inhibitor meloxicam through the screening of the FDA drug library.

As a popular antitumor chemotherapy regimen in recent years, PD1 has achieved satisfactory clinical results in the treatment of advanced HCC. However, the problems of PD1 monoclonal antibody resistance in further treatment have also triggered our thinking. Starting from the results of drug screening, we further confirmed the antitumor effect of meloxicam in animal experiments, especially the impressive therapeutic effect after combined with PD1 monoclonal antibody.

Previous studies have shown that microRNA-200 is a cell-autonomous inhibitor of epithelial-to-mesenchymal transition (EMT) and metastasis and can target PD-L1 to inhibit its expression. ZEB1 can activate EMT inhibited by microRNA-200 and break the targeting relationship between microRNA-200 and PDL1 [24]. Studies have confirmed that downregulation of microRNA-200 can upregulate the expression of PD-L1 in breast cancer [12]. In order to further explain the excellent therapeutic effect of meloxicam combined with PD1 monoclonal antibody on mouse HCC models, we discovered through a series of experiments that COX2 can interact with microRNA-200 to cause changes in PD-L1 levels. Our experimental results confirmed that after knocking down the expression of COX2, microRNA-200 in tumor cells decreased significantly, which may be a mechanism for meloxicam to upregulate the level of PD-L1.

Therefore, our experiments confirmed that meloxicam can inhibit the proliferation, invasion, and migration of different HCC cell lines. The novelty is that, for the first time, we screened meloxicam, a drug with strong anti-HCC potential, from 800 FDA-approved small-molecule drugs. We demonstrate for the first time that meloxicam remodels PD-L1 expression and enhances tumor immunotherapy sensitivity through the miR-200/PD-L1 pathway. We also demonstrated that in in vivo experiments in mice, meloxicam combined with PD1 monoclonal antibody is more effective than traditional monotherapy in the treatment of HCC, and it has a very strong clinical application potential for PD1-resistant patients. However, our experiment also has many imperfections. We did not use the patient-derived xenograft model to further verify our conclusions. At the same time, we did not coculture the cancer cells used in the experiment with CD8^+^ T cells to more accurately simulate the HCC tumor microenvironment.

## 5. Conclusion

Meloxicam inhibits hepatocellular carcinoma progression and enhances the sensitivity of immunotherapy via the microRNA-200/PD-L1 pathway.

## Data Availability

All the data are included within the article.

## Supplementary Materials

Supplementary Materials In order to screen new drugs with anti-HCC efficacy, we used the CCK8 method to determine more than 800 drugs against the YY8103 cell line, and the experimental result is in Supplementary Table 1.
