# Supplementary material for: Meloxicam Inhibits Hepatocellular Carcinoma Progression and Enhances the Sensitivity of Immunotherapy via the MicroRNA-200/PD-L1 Pathway
Source: J Oncol. 2022 Feb 21;2022:4598573. doi: 10.1155/2022/4598573 (PMC8885196; doi:10.1155/2022/4598573)
Supplement: Supplementary Materials — In order to screen new drugs with anti-HCC efficacy, we used the CCK8 method to determine more than 800 drugs against the YY8103 cell line, and the experimental result is in Supplementary Table 1. [file 4598573.f1.pdf]

Supplementary Table 1 Drug names from selleck drug library approved by FDA

S4656	S1214	S5325	S3747	S5004	S2596	S1005	S8041	S4820	S5776	S1646
Parecoxib	Bleomycin sulfate	Nitisinone	Levothyroxine sodium	Pimecrolimus	Clindamycin palmitate HCl	Axitinib	Cobimetinib (GDC-0973, RG7420)	Diastase	Proflavine	Ketorolac
S4657	S1218	S5326	S3750	S5005	S2599	S1007	S8048	S4830	S5778	S1647
Eslicarbazepine Acetate	Clofarabine	Dolasetron	Sodium benzoate	Cefotiam hydrochloride	L-Thyroxine	Roxadustat (FG-4592)	Venetoclax (ABT-199, GDC-0199)	Maltose	Esmolol	Adenosine
S4658	S1221	S5327	S3751	S5006	S2601	S1010	S8051	S4831	S5779	S1649
Hydroquinidine	Dacarbazine	Meisoindigo	Quinidine sulfate	Teprenone	Gliclazide	Nintedanib (BIBF 1120)	Macitentan	Piperonyl butoxide	Trimetazidine	Zolmitriptan
S4660	S1222	S5328	S3754	S5007	S2602	S1011	S8067	S4832	S5780	S1651
Glycopyrrolate	Dexrazoxane HCl (ICRF-187, ADR-529)	Gamithromycin	4-Hydroxybenzoic acid	Delamanid	Acemetacin	Afatinib (BIBW2992)	Vorapaxar	Tolmetin	Prazosin	Telbivudine
S4661	S3061	S5331	S3755	S5009	S2603	S1013	S8101	S4833	S5781	S1652
Tiagabine hydrochloride	Epinephrine HCl	Ceftezole sodium	Betaine	Brivudine	Tioxolone	Bortezomib (PS-341)	CB-5083	Cefoxitin sodium	Raloxifene	Monobenzone
S4662	S3062	S5333	S3756	S5010	S2604	S1014	S8116	S4834	S5782	S1653
Atazanavir	Diclofenac Potassium	Sulbenicillin Sodium	Methyl salicylate	Indometacin Sodium	Dehydroepiandrosterone (DHEA)	Bosutinib (SKI-606)	Acalabrutinib (ACP-196)	Propantheline bromide	Doxazosin	Tretinoin
S4663	S3063	S5341	S3758	Empty	S2605	S1021	S8133	S4835	S5783	S1654
Fusidate Sodium	Diclofenac Diethylamin	Metroprolol succinate	Sinomenine hydrochloride		Idebenone	Dasatinib	Resiquimod	Aceclofenac	Montelukast	Phenylbutazone

	e									
S4664	S3066	S5343	S3761	S5012	S2606	S1022	S8134	S4836	S5784	S1655
Molsidomine	Naloxone HCl	Vanillic acid	Eucalyptol	Octenidine Dihydrochloride	Mifepristone	Ridaforolimus (Deforolimus, MK-8669)	Radotinib	Nilutamide	Vancomycin	Ezetimibe
S4665	S3067	S5345	S3763	S5013	S2607	S1025	S8135	S4837	S5787	S1657
Rebeprazole sodium	Chlorhexidine?2HCl	Nerolidol	Cinnamaldehyde	Ioversol	Buflomedil HCl	Gefitinib (ZD1839)	Riociguat (BAY 63-2521)	Ibudilast	Dronedarone	Enalaprilat Dihydrate
S4666	S3070	S5350	S3766	S5014	S2608	S1026	S8136	S4838	S5795	S1658
Sivelestat sodium tetrahydrate	Piracetam	Cefpodoxime proxetil	Tanshinone IIA sulfonate (sodium)	Crisaborole (AN2728)	Fluocinonide	Imatinib Mesylate (STI571)	Sivelestat (ONO-5046)	Acotiamide hydrochloride	Rasagiline	Dofetilide
S4667	S3071	S5351	S3769	S5015	S2609	S1028	S8144	S4839	S5802	S1662
Lidocaine hydrochloride	Vanillin	Cefmetazole sodium	Palmatine	Simeprevir	Inulin	Lapatinib (GW-572016) Ditosylate	Halofuginone	Mosapride	Alprenolol hydrochloride	Isradipine
S4668	S3074	S5352	S3772	S5016	S2610	S1029	S8146	S4841	S5805	S1665
Procaine	Chlorthalidone	Cefminox Sodium	5-Hydroxymethylfurfural	Isoprinosine	Lonidamine	Lenalidomide (CC-5013)	Mitomycin C	Laurocapram	Allopregnanolone	Estrone
S4669	S3075	S5353	S3773	S5017	S2613	S1030	S8183	S4843	S5808	S1666
Benzocaine hydrochloride	Dexmedetomidine	Cefpiramide sodium	Tyrosol	Oxyclozanide	Clorsulon	Panobinostat (LBH589)	Pimavanserin	Potassium acetate	Relugolix	Flucytosine
S4673	Empty	S5356	S3775	S5019	S2614	S1033	S8195	S4844	S5812	S1667

Etonogestrel		Ceftiofur	Ligustrazine hydrochloride	Indobufen	Arecoline HBr	Nilotinib (AMN-107)	Oclacitinib?maleate	Cefcapene Pivoxil Hydrochloride	Choline Fenofibrate	Trichlormethiazide
S4674	S3077	S5357	S3779	S5020	S2615	S1035	S8205	S4845	S5816	S1669
Hydroxyprogesterone caproate	Tazobactam	Safinamide	cis-Anethole	Tilorone dihydrochloride	Noradrenaline bitartrate monohydrate	Pazopanib HCl (GW786034 HCl)	Enasidenib (AG-221)	Rabeprazole	pyrvinium	Loteprednol etabonate
S4675	S3078	S5358	S3781	S5023	S2625	S1039	S8206	S4846	S5821	
Tiagabine	Beclomethasone dipropionate	Regadenoson	Ginkgolide C	Nadolol	Fostamatinib (R788)	Rapamycin (Sirolimus)	Ivosidenib (AG-120)	Meropenem Trihydrate	Linoleic acid	Empty
S4676	S3079	S5359	S3783	S5025	S2664	S1040	S8266	S4847	S5827	S1672
Gluconolactone	Atovaquone	Butoconazole	Echinacoside	Efinaconazole	Clinofibrate	Sorafenib Tosylate	Melphalan	Faropenem Sodium	Citronellyl acetate	Aminoglutethimide
S4678	S3080	S5360	S3785	S5027	S2665	S1042	S8294	S4848	S5842	S1673
Povidone iodine	Etravirine (TMC125)	Diflorasone	Notoginsenoside R1	Mebeverine Hydrochloride	Ciprofibrate	Sunitinib Malate	Olmutinib (HM61713, BI 1482694)	Dalbavancin	Cabergoline	Aminophylline
S4679	S3081	S5361	S3788	S5028	S2667	S1044	S8401	S4849	S5843	S1675
Terazosin HCl	Ulipristal	Bendazac	Carvacrol	4-Aminopyridine	Dolutegravir (GSK1349572)	Temsirolimus (CCI-779, NSC 683864)	Erdafitinib (JNJ-42756493)	Levocetirizine Dihydrochloride	Cinitapride Hydrogen Tartrate	Lubiprostone
S4680	S3083	S5363	S3791	S5029	S2673	S1047	S8432	S4850	S5858	S1676

Protirelin	Indacaterol Maleate	Pikamilone	Succinic acid	Etofylline	Trametinib (GSK1120212)	Vorinostat (SAHA, MK0683)	Troglitazone (CS-045)	Flucloxacillin sodium	Vilazodone	Amorolfine HCl
S4682	S3100	S5365	S3794	S5030	S2680	S1053	S8539	S4851	S5861	S1677
Loxoprofen	2-Thiouracil	Alogliptin	Palmitic acid	Dihydralazine sulphate	Ibrutinib (PCI-32765)	Entinostat (MS-275)	TAS-102	Tafluprost	Lercanidipine	Chloramphenicol
S4683	S3104	S5366	S3802	S5032	S2721	S1055	S8558	S4852	S5862	S1679
Sildenafil Mesylate	Moguisteine	Fipronil	Trigonelline Hydrochloride	Mephenesin	Nilvadipine	Enzastaurin (LY317615)	Tofogliflozin(CSG 452)	Gadopentetate Dimeglumine	Metoclopramide	Flurbiprofen
S4685	S3105	S5367	S3805	S5033	S2727	S1060	S8565	S4853	S5863	S1680
Efavirenz	Nadifloxacin	Ethyl Oleate	Stevioside	Terconazole	Dacomitinib (PF299804, PF299)	Olaparib (AZD2281, Ku-0059436)	Omarigliptin (MK-3102)	Ecabet sodium	Metronidazole Benzoate	Disulfiram
S4686	S3106	S5368	S3807	S5034	S2741	S1064	S8567	S4854		S1681
Vitamin E	Pidotimod	Lactitol	Dehydroandrographolide	Melitracen hydrochloride	Niraparib (MK-4827)	Masitinib (AB1010)	Tucidinostat (Chidamide)	Bedaquiline fumarate	Empty	Mesalamine
S4687	S3113	S5369	S3809	S5037	S2760	S1068	S8594	S4856	S5869	S1683
Rivastigmine	Pyridoxine HCl	Ethoxyquin	Imperatorin	Phenazine methosulfate	Canagliflozin	Crizotinib (PF-02341066)	Tirofiban Hydrochloride	Iproniazid	Cortisone	Ipratropium Bromide
S4689	S3114	S5371	S3810	S5038	S2762	S1082	S8615	S4859	S5871	S1685
Deoxycholic acid	Vitamin C	Ajmaline	Scutellarin	Valethamate Bromide	Alectinib (CH5424802)	Vismodegib (GDC-0449)	Sodium dichloroacetate (DCA)	TriacetonaMine	Trans-Tranilast	Sulfanilamide
S4690	S3116	S5372	S3811	S5039	S2765	S1085	S8637	S4860	S5872	S1688

Escin	Sulfathiazole	Methyl Aminolevulinate Hydrochloride	Ginsenoside Re	Actarit	MK-2048	Belinostat (PXD101)	Ipragliflozin (ASP1941)	Indole-3-carboxylic acid	Estradiol dipropionate(17-Beta-Estradiol-3,17-Dipropionate)	Betamethasone Dipropionate
S4691	S3117	S5377	S3817	S5040	S2787	S1098	S8726	S4862	S5873	S1689
Oxybenzone	Oxybutynin chloride	Dibutyl phthalate	Harmine hydrochloride	Tiamulin	Laquinimod	Rucaparib (AG-014699,PF-01367338) phosphate	Anlotinib (AL3818) dihydrochloride	Squalene	Scopolamine HBr trihydrate	Meprednisone
S4693	S3120	S5378	S3824	S5041	S2789	S1119	S9001	S4865	S5874	S1690
Guanfacine Hydrochloride	Doxepin HCl	Dimethyl phthalate	Quercitrin	Difloxacin hydrochloride	Tofacitinib (CP-690550,Tasocitinib)	Cabozantinib (XL184, BMS-907351)	Malic acid	Cefetamet pivoxil hydrochloride	L-Carnitine hydrochloride	Betamethasone Valerate
S4695	S3121	S5382	S3835	S5042	S2790	S1120	S9002	S4866	S5900	S1691
D panthenol	Ornidazole	Formate	Loganin	Bevantolol hydrochloride	Istradefylline	Everolimus (RAD001)	L-Fucose	Nicarbazin	Edrophonium chloride	Praziquantel
S4696	S3124	S5385	S3842	S5043	S2792	S1137	S9003	S4867	S5901	S1692
CarbinoxaMine Maleate	Dexamethasone Acetate	Imidafenacin	Isoquercitrin	Benorylate	Torcetrapib	Malotilate	(R)-(-)-Mandelic acid	Propacetamol hydrochloride	Canagliflozin hemihydrate	Busulfan
S4697	S3129	S5388	S3843	S5046	S2794	S1144	S9007	S4868	S5909	S1693
Saxagliptin hydrate	Trimethoprim	Betrixaban	Madecassoside	Clonixin	Sofosbuvir (PSI-7977,	Ivacaftor (VX-770)	2'-Deoxyguanosine monohydrate	Xanthinol Nicotinate	Anagliptin	Carbamazepine

					GS-7977)					
S4698	S3130	S5389	S3847	S5049	S2807	S1148	S9032	S4869	S5911	S1696
Vitamin K1	Biotin (Vitamin B7)	Betrixaban maleate	Panaxatriol	Thiocolchicoside	Dabrafenib (GSK2118436)	Docetaxel	Sanguinarine	Sulfamethoxazole sodium	Bictegravir	Hydrocortisone
S4699	S3132	S5392	S3849	S5052	S2809	S1150	S9042	S4870	S5923	S1701
Etretinate	Sulfamerazine	Mepivacaine	D-Galactose	Granisetron	MPEP	Paclitaxel	Wedelolactone	Cefodizime Sodium	Celiprolol hydrochloride	Desonide
S4701	S3133	S5393	S3850	S5054	S2814	S1156	S9046	S4871	S5924	S1702
2-Deoxy-D-glucose	Sulfamethazine	Cyclofenil	Glucosamine sulfate	Rifamycin sodium salt	Alpelisib (BYL719)	Capecitabine	Berberine	Pyridoxal 5-phosphate monohydrate	Olanexidine Hydrochloride semihydrate	Didanosine
S4706	S3137	S5395	S3851	S5055	S2830	S1164	S9063	S4874	S5925	S1703
Eugenol	Sodium salicylate	Phenolphthalein	Camphor	Milbemycin Oxime	Clindamycin	Lenvatinib (E7080)	Harringtonine	Cefazedone	Olodaterol hydrochloride	Divalproex Sodium
S4707	S3138	S5397	S3854	S5056	S2832	S1168	S9140	S4875	S5926	S1704
Oleic Acid	Methylthiouracil	Chlorhexidine	Tetrahydropalmatine hydrochloride	Dinoprost tromethamine	Epiandrosterone	Valproic acid sodium salt (Sodium valproate)	Pulegone	Cephapirin Benzathine	Pitolisant hydrochloride	Emtricitabine
S4709		S5398	S3856	S5058	S2840	S1178	S9141	S4876	S5927	S1705
Latanoprost	Empty	Nefazodone hydrochloride	Allantoin	Revaprazan Hydrochloride	Apalutamide?(ARN-509)	Regorafenib (BAY 73-4506)	Berbamine	Robenidine Hydrochoride	Proguanil	Progesterone
S4711	S3140	S5399	S3858	S5059	S2851	S1183	S9142	S4877	S5935	S1706

Esculetin	Milnacipran HCl	Chlorprothixene hydrochloride	Lawsone	Pixantrone Maleate	Baricitinib (LY3009104, INCB028050)	Danoprevir (ITMN-191)	Sparteine	Eperisone hydrochloride	Alvimopan	Lamivudine
S4714	S3144	S5401	S3866	S5060	S2853	S1185	S9143	S4878	S5950	S1709
(-)-Menthol	Darifenacin HBr	Tegaserod Maleate	Galanthamine	Metadoxine	Carfilzomib (PR-171)	Ritonavir	Ammonium Glycyrrhizate	Neticonazole Hydrochloride	Fingolimod	Estradiol
S4716	S3147	S5402	S3868	S5062	S2868	S1188	S9193	S4880	S6003	S1712
Evans Blue	Entacapone	Dasabuvir(ABT-333)	Harmine	Daclatasvir Digydrochloride	Alogliptin （SYK-322） benzoate	Anastrozole	Aristolochic acid A	Cefathiamidine	Ataluren (PTC124)	Deferasirox
S4717	S3149	S5403	S3872	S5063	S2874	S1189	S9212	S4881	S6006	S1713
Isatin	Estradiol valerate	Ombitasvir (ABT-267)	Guaiacol	Trelagliptin succinate	Camostat Mesilate	Aprepitant	Melamine	Calcium Dobesilate	Imidazole	Piroxicam
S4718	S3150	S5404	S3876	S5065	S2875	S1190	S9222	S4883	S6007	S1714
Acetylcholine iodide	Articaine HCl	Paritaprevir (ABT-450)	Indigo	Ganciclovir sodium	Prucalopride	Bicalutamide	Dipotassium glycyrrhizinate	Lynestrenol	Bisphenol A	Gemcitabine
S4722	S3151	S5405	S3881	S5066	S2884	S1191	S9227	S4885	S6010	S1715
(+)-Catechin	Gliquidone	Propylparaben	Scopoletin	Pramipexole dihydrochloride	Acesulfame Potassium	Fulvestrant	Sinensetin	Taurolidine	Sodium L-lactate	Glipizide
S4723	S3154	S5406	S3883	S5067	S2900	S1192	S9240	S4886	S6014	S1716
(-)Epicatechin	Butenafine HCl	Sultamicillin Tosylate	Protopine	Losartan	Cobicistat (GS-9350)	Raltitrexed	Isofraxidin	Menbutone	Isonicotinic acid	Glyburide (Glibenclamide)
S4725	S3155	S5407	S3885	S5069	S2902	S1193	S9249	S4887	S6017	S1717

Benzenesulfonamide	Mepivacaine HCl	Squalane	Pyrogallol	Dabrafenib Mesylate	S-Ruxolitinib (INCB018424)	Thalidomide	Securinine	Nikethamide	p-Cresol	Fomepizole
S4726	S3160	S5408	S3887	S5070	S2903	S1196	S9263	S4889	S6034	S1718
Lauric Acid	Ethynodiol diacetate	Isoprene	L-Rhamnose monohydrate	Mupirocin calcium	Lumiracoxib	Exemestane	3-n-Butylphathlide	Perospirone hydrochloride	p-Benzoquinone	Adefovir Dipivoxil
S4727	S3161	S5410	S3889	S5071	S2907	S1197	S9311	S4890	S6035	S1719
Cinnarizine	Sertaconazole nitrate	Chloramphenicol sodium succinate	Arteether	Duloxetine	Pirfenidone	Finasteride	Germacrone	Bifendate	2-Naphthol	Zalcitabine
S4731	S3162	S5411	S3892	Empty	S2922	S1199	S9321	S4893	S6041	S1721
Perphenazine	Tylosin tartrate	Sultamicillin	Isopsoralen		Icotinib	Cladribine	Topotecan	Cytosine	Dibutyl sebacate	Azathioprine
S4733	S3163	S5413	S3893	S5073	S3000	S1200	S9326	S4896	S6047	S1723
Retigabine	Benztropine mesylate	Ertugliflozin	Bornyl acetate	Donepezil	Carbazochrome sodium sulfonate (AC-17)	Decitabine	Scopolamine	Elagolix Sodium	Lactose	Indomethacin
S4734	S3167	S5414	S3895	S5074	S3001	S1202	S9327	S4899	S6049	S1725
Retigabine 2HCl	Altrenogest	Diflucortolone valerate	Sophoridine	Argatroban Monohydrate	Clevudine	Dutasteride	Carboprost	Sulfogaiacol	(+)-(S)-Carvone	Terbinafine
S4735	S3170	Empty	S3898	S5075	S3002	S1204	S9329	S4931	S6052	S1727
Salvianolic acid B	Ampicillin sodium		Hydroxy Camptothecine	Acotiamide	Rivaroxaban	Melatonin	Orcinol	Propiverine hydrochloride	Saccharin sodium salt hydrate	Levonorgestrel
S4736	S3172	S5416	S3899	S5076	S3003	S1206	S9338	S4932	S6053	S1729

Trapidil	Anagrelide HCl	Metyrapone	Hederagenin	Xipamide	Prostaglandin E2 (PGE2)	Bisoprolol fumarate	Octyl gallate	Proxyphylline	Phenylglyoxylic acid	Gemfibrozil
S4737	S3173	S5418	S3901	S5077	S3005	S1207	S9346	Empty	S6055	S1730
Psoralen	Antipyrine	Parecoxib Sodium	Astragaloside IV	Regorafenib Monohydrate	Paroxetine HCl	Tivozanib (AV-951)	1-Indanone		(S)-(–)-Limonene	Indapamide
S4748	S3175	S5419	S3909	S5078	S3008	S1208	S9349	S4935	S6058	S1732
Ondansetron Hydrochloride Dihydrate	Atomoxetine HCl	1,4-Cineole	Catalpol	Osimertinib mesylate	Zaltoprofen	Doxorubicin (Adriamycin) HCl	D-(+)-Raffinose pentahydrate	Asunaprevir	β-Caryophyllene	Mitotane
S4749	S3176	S5420	S3914	S5079	S3012	S1209	S9351	S4939	S6067	S1733
Citalopram HBr	Betahistine 2HCl	Clindamycin alcoholate	α-Hederin	Sitagliptin	Pazopanib	Fluorouracil (5-Fluoracil, 5-FU)	2'-Deoxyadenosine monohydrate	cis-Aconitic acid	2,4-dichlorobenzyl alcohol	Methylprednisolone
S4750	S3178	S5424	S3919	S5081	S3015	S1210	S9354	S4940	S6068	S1734
Sulfacetamide sodium salt hydrate	Brinzolamide	Carbaryl	Hederacoside C	Ceforanide	Amoxicillin	Methotrexate	Oxalic acid	Maltol	Doxycycline monohydrate	Meloxicam
S4751	S3179	S5428	S3921	S5082	S3017	S1212	S9368	S4949	S6073	S1735
Cisapride hydrate	Carbenicillin disodium	Promazine hydrochloride	Lathyrol	Vitamin K2	Aspirin	Bendamustine HCl	ADP	Nonanoic acid	Diclofenac acid	Mesna
S4752	S3180	S5430	S3923	S5083	S3018	S1223	S9373	S4952	S6079	S1736
Corticosterone	Eletriptan HBr	Metoprolol	Ginsenoside Rg1	Lentinan	Niflumic acid	Epirubicin HCl	Neryl acetate	Fumaric acid	2-Methylhexanoic acid	Methocarbamol
S4754	S3181	S5435	S3924	S5084	S3019	S1225	S9380	S4953	S6104	S1737

Betulin	Flumequine	Quinacrine Dihydrochloride Dihydrate	Ginsenoside Rb1	Carbazochrome	Ciclopirox ethanolamine	Etoposide	5,7-Dihydroxy-4-methylcoumarin	Usnic acid	(±)-α-Tocopherol	Prednisolone
S4757	S3183	S5440	S3925	S5085	S3021	S1227	S9382	S4957	S6118	S1738
Dihydrotestosterone(DHT)	Amitriptyline HCl	Berberine Sulfate	(-)-Epicatechin gallate	Azamethiphos	Rimonabant	Raloxifene HCl	Thymine	Linalool	Terpinen-4-ol	Telmisartan
S4759	S3185	S5447	S3926	S5086	S3022	S1229	S9383	S4958	S6121	S1739
p-Coumaric Acid	Adrenalone HCl	Triprolidine Hydrochloride	Forsythin	p-Anisaldehyde	Cabazitaxel	Fludarabine Phosphate	Methyl palmitate	Glycocholic acid	Maltotriose	Thiabendazole
S4768	S3186	S5450	S3927	S5087	S3023	S1231	S9385	S4964	S6172	S1740
Melibiose	Azatadine dimaleate	Sofalcone	Swertiamarin	Tianeptine	Bufexamac	Topotecan HCl	Ligustilide	Lactobionic acid	Brucine sulfate heptahydrate	Guaifenesin
S4769	S3188	S5452	S3930	S5091	S3024	S1233	S9400	S4971	S6173	S1741
L-5-Hydroxytryptophan	(+,-)-Octopamine HCl	Sanguinarine chloride	Liquiritin	Geranyl acetate	Lamotrigine	2-Methoxyestradiol (2-MeOE2)	Rhynchophylline	Buparvaquone	Amylmetacresol	Rifabutin
S4776	S3189	S5453	S3935	S5092	S3027	S1235	S9413	S4973	S6188	S1742
Harmaline	Ropinirole HCl	Hyperoside	Nonivamide	Febantel	Fenoprofen calcium hydrate	Letrozole	Yangonin	Iminostilbene	p-Toluenesulfonic acid monohydrate	Nevirapine
S4779	S3193	S5454	S3944	S5093	S3031	S1237	S9451	S4975	S6196	S1743
Menadiol Diacetate	Ticarcillin sodium	Saikosaponin D	Valproic acid	Rafoxanide	Linagliptin	Temozolomide	Uridine 5'-monophosphate	Fimasartan	4-Nitrophenol	NEXIUM (esomeprazole magnesium)
S4783	S3195	S5457	S3945	S5096	S3033	S1238	S9454	S4976	S6197	S1744

Benzyl isothiocyanate	Azlocillin sodium salt	Curculigoside	L-Cycloserine	SulfadiMethoxine sodium	Vildagliptin (LAF-237)	Tamoxifen	Undecanoic acid	Sulfalene(SMPZ)	Dimethylamine hydrochloride	Nicotinic Acid
S4792	S3199	S5459	S3946	Empty	S3035	S1241	S9457	S4977	S6199	S1747
N-Acetylneuraminic acid	Reboxetine mesylate	Aucubin	Mesterolone		Daunorubicin HCl	Vincristine sulfate	Phensuximide	Efonidipine	o-Cresol	Nimodipine
S4794	S3200	S5466	Empty	S5100	S3036	S1243	S9458	S4978	S6202	S1748
Drostanolone Propionate	Triflusal	Saikosaponin A		Pralidoxime Iodide	Pravastatin sodium	Agomelatine	Ergoloid Mesylates	Azathramycin	Butylated hydroxytoluene	Nisoldipine
S4795	S3201	S5473	S3950	S5102	S3037	S1247	S9459	S4980	S6207	S1750
Trenbolone acetate	Trifluoperazine 2HCl	Pivmecillinam hydrochloride	Maltitol	Stachyose	Bepotastine Besilate	Leflunomide	Mecamylamine Hydrochloride	Anamorelin	Levulinic acid	Octocrylene
S4796	S3202	S5476	S3951	S5103	S3038	S1250	S9460	S4983	S6210	S1754
Methandrostenolone	Catharanthine	Rolapitant	Tannic acid	lutein	Fosaprepitant dimeglumine salt	Enzalutamide (MDV3100)	Ethotoin	Sorbic acid	Ethanolamine hydrochloride	Oxybutynin
S4797	S3204	S5477	S3957	S5105	S3043	S1251	S9461	Empty	S6211	S1756
Nicergoline	Meptazinol HCl	Gefarnate	Gamma-Oryzanol	Proanthocyanidins	Rofecoxib	Dienogest	Benzonatate		Furfural	Enoxacin
S4800	S3207	S5478	S3959	S5106	S3045	S1252	Empty	S4988	S6215	S1759
Daminozide	Iopromide	Dantrolene sodium	(+)-Borneol	Lanolin	Cinepazide maleate	Entecavir Hydrate		Tiamulin fumarate	Phthalic acid	Pitavastatin Calcium
S4803	S3208	S5479	S3965	S5108	S3047	S1255	S9463	S4991	S6217	S1760

Thymidine	Fexofenadine HCl	Cloperastine hydrochloride	Vanillyl Butyl Ether	Tylosin	Otilonium Bromide	Nepafenac	Demecarium Bromide	Valpromide	o-Toluic acid	Rifapentine
S4812	S3212	S5480	S3966	S5109	S3051	S1256	S9465	S4994	S6218	S1761
Ceftizoxime	Moclobemide (Ro 111163)	Clidinium Bromide	Nifuratel	Ademetionine	Bosentan Hydrate	Rufinamide	Methysergide Maleate	Methylcobalamin	Sodium lauryl sulfate	Suprofen
S4813	S3604	S5481	S3967	S5112	S3052	S1257	S9466	S4996	S6221	S1762
Cefuroxime axetil	Triptolide (PG490)	Molindone hydrochloride	Flavone	alpha-Arbutin	Rupatadine Fumarate	Posaconazole	Methenamine Hippurate	Tavaborole (AN-2690)	Methyl cinnamate	Pyrazinamide
S4815	S3605	S5482	S3968	S5113	S3053	S1258	S9467	S4999	S6223	S1763
L-Cysteine HCl	Borneol	Prilocaine hydrochloride	Histamine	Propyl gallate	Azelnidipine	Prasugrel	Thiothixene	Avermectin B1(Abamectin)	Triethyl citrate	Quetiapine Fumarate
S4816	S3606	S5483	S3969	S5114	S3054	S1259	S9469	S5001	S6226	S1764
Diatrizoate sodium	Fangchinoline	Tribenzagan Hydrochloride	Veratric acid	Hydroquinine	Alverine Citrate	Ramelteon	Haloperidol Decanoate	Tofacitinib (CP-690550) Citrate	4-Methyl-2-pentanone	Rifampin
S4817	S3609	S5484	S3970	S5117	S3057	S1260	S9470	S5002	S6231	S1768
Atenolol	Berbamine (dihydrochloride)	Rimantadine Hydrochloride	Vindoline	Doramectin	Azilsartan Medoxomil	Cinacalcet HCl	Penbutolol Sulfate	Fingolimod (FTY720) HCl	Methyl nicotinate	Cefditoren Pivoxil
S4819	S3611	S5485	S3971	S5119	S3060	S1261	S9472	S5003	S6233	S1770

Saccharin	(+)-Fangchinoline	Desipramine Hydrochloride	Fusidine	Olivetol	Medetomidine HCl	Celecoxib	Oxtriphylline	Tacrolimus (FK506)	Tartaric acid	Sulfadiazine

Empty means no drug in this hole.
